# The Determination of the Prohibited Herbicide 4,6-Dinitro-Ortho-Cresol (DNOC) in Poisoned Domestic and Wild Animals in Italy

**DOI:** 10.3390/ani14172483

**Published:** 2024-08-26

**Authors:** Rachele Rocchi, Antonella Tinelli, Giuseppe Gatti, Pietro Badagliacca, Antonio Cocco, Maria Chiara Cantelmi, Antonella Damiano, Giampiero Scortichini, Carmine Merola, Antonio Petrini

**Affiliations:** 1 Istituto Zooprofilattico Sperimentale dell’Abruzzo e Molise “G. Caporale”, Campo Boario, 64100 Teramo, Italy; r.rocchi@izs.it (R.R.); g.gatti@izs.it (G.G.); piba@live.it (P.B.); a.cocco@izs.it (A.C.); m.cantelmi@izs.it (M.C.C.); g.scortichini@izs.it (G.S.); a.petrini@izs.it (A.P.); 2 Department of Veterinary Medicine, University of Bari “Aldo Moro”, Valenzano, 70010 Bari, Italy; antonella.tinelli@uniba.it; 3 Department of Bioscience and Technology for Food, Agriculture and Environment, University of Teramo, 64100 Teramo, Italy; adamiano@unite.it

**Keywords:** poison baits, pesticides, acute poisoning, animal health, environmental health

## Abstract

**Simple Summary:**

This study reported for the first time in Italy the determination of 4,6-dinitro-ortho-cresol (DNOC) in the baits and gastric contents of poisoned animals. The determination of DNOC was achieved by using gas chromatography coupled to mass spectrometry (GC-MS) with both full-scan and selected ion monitoring (SIM) modes (ion *m*/*z* values 198, 121, and 105).

**Abstract:**

This study reports the determination of DNOC in the baits and gastric contents of poisoned dogs and wild canids collected in the Abruzzo region between 2014 and 2022. A total of 663 samples of the baits and carcasses of domestic and wild animals were analyzed for the determination of DNOC through GC-MS. DNOC was found to be present in 58 cases from 663 analyzed samples, with 49 poison baits and nine gastric content samples from dogs and red foxes. This study confirms for the first time that the banned pesticide DNOC still poisons both domestic and wild animals in the Abruzzo region. It should be considered that this study was carried out on a population of animals from a regional geographical area, and more detailed country-wide studies need to be carried out to obtain the incidence of poisoning from this herbicide in Italy. Furthermore, these findings emphasize the importance of considering this chemical in differential diagnosis during toxicological investigations of animal poisoning.

## 1. Introduction

The intentional, illegal, or accidental poisoning of both domestic and wild animals has been described by many authors worldwide. Intoxication can be caused by several compounds, but pesticides are the most frequently responsible in Europe and Italy [1]. Pesticides are used to protect crops and improve production yields by reducing losses due to insect pests, plant pathogens, and weeds [2,3,4]. The incidence of poisonings related to pesticides is influenced by many factors, such as the availability on the local, international, and online market, agricultural techniques, and cultural background [5]. Intoxications can result from an incorrect use or abuse of these compounds from poor management to intentional poisoning, even with baits, of species considered harmful for human activities but also of domestic animals [6]. The pesticides most frequently involved in animal poisoning in Italy and also in Europe are insecticides, molluscicides, and rodenticides [7,8]. Poisoning episodes by herbicides and fungicides have also been reported but less frequently [7]. In fact, herbicides such as diquat and paraquat have been sporadically involved in poisoning animals [9].

Despite the European ban on the use of highly toxic pesticides, poisonings involving aldicarb, carbofuran, diazinon, endosulfan, and fenthion are still frequently reported in domestic animals and wildlife [10]. This is likely due to the use of personal supplies or the illegal trade of prohibited chemicals. A study conducted in Spain [11] found that aldicarb and carbofuran were present in nearly 75% of poisoning cases, with the percentage nearing 100% when considering the analyzed baits. The availability of restricted substances presents significant diagnostic and clinical challenges that must be addressed to reduce the incidence of poisonings. In this context, the continuous and accurate collection of epidemiological data on animal poisonings remains essential. These data offer valuable insights into toxicant trends, the emergence of new substances, and the re-emergence of previously restricted compounds. Such information can assist veterinarians in managing suspected poisoning cases and help health authorities improve preventive measures for effective risk management, thereby protecting animal, human, and environmental health.

In Italy, to safeguard the health of people, animals, and the environment, the possession, improper use, preparation, mixing, and scattering of poison baits are prohibited, as established by a Decree of the Ministry of Labor, Health, and Social Policies. This Decree has been in effect since January 2009 [12].

Enhancing knowledge about poisoning from banned herbicides could improve the collection of epidemiological data and ensure that new confirmed cases involving “old” substances are properly documented. This could also help ensure the inclusion of these chemicals in clinical and laboratory processes for accurate diagnosis.

The aim of this article is to describe, for the first time in Italy, the detection of 4,6-dinitro-ortho-cresol (DNOC), a banned pesticide, in the baits and carcasses of suspected poisoned domestic and wild animals collected in the Abruzzo region using GC-MS analysis.

## 2. Materials and Methods

### 2.1. History

In early December 2014, in a limited area dedicated to truffle production between the Pescara and Chieti provinces (Abruzzo region, Italy), a massive poisoning involving four dogs and a red fox was associated with the recovery of at least 22 baits consisting of meatballs composed of minced truffle and yellow-greenish-colored meat. The same material was detected in the composition of the stomach contents of poisoned animals. All toxicological analyses routinely carried out by the laboratory including pesticides, pyrethroids, and carbamates commonly used in agriculture, metaldehyde, strychnine, and rodenticides were tested negative. An unknown spectrometric signal was observed in these samples and identified for the first time as 4,6-dinitro-ortho-cresol (DNOC). Subsequently, sporadic events of animal poisoning and the recovery of baits poisoned by DNOC occurred in the same territory until late 2020, when a yellow powder was seized from a private home and identified by the laboratory as DNOC. Analytical determinations of DNOC were performed individually between 2014 and 2020 according to the suspected results of anatomopathological investigation for carcasses and visual inspection for poison baits. In 2021, DNOC was included in a multi-residue analytical method for determining pesticides.

### 2.2. Sample Collection

Between 2014 and 2022, samples including animal carcasses and baits suspected of poisoning were submitted by local veterinarians to the “Istituto Zooprofilattico Sperimentale Abruzzo e Molise” (IZSAM) in accordance with ministerial guidelines. Data on the recovery site and individual information about the animals (such as species and geographic coordinates) were also recorded. The first diagnostic step involved necropsy for carcasses and visual inspection for baits. Animals for which other causes of death were confirmed, such as traumatic lesions and infectious diseases, were not considered potential poisoning cases. However, partially confirmed or still-suspected carcasses and baits were sent for further analytical procedures. Toxicological investigations were conducted on both domestic and wild animal carcasses and poison baits. The determination of DNOC was performed on 663 samples, which included 180 baits, 270 gastric content samples, 34 intestinal content samples, and 179 liver samples (*n* = 663). The collection of biological matrices, in addition to the 180 baits, included 191 samples from dogs, 37 from red foxes, 108 from cats, 81 from wild birds, 45 from other wild carnivores, 17 from farm animals, and 4 from other animals (*n* = 663). Samples were collected from the Abruzzo and Molise regions.

### 2.3. Analysis

Poison baits and biological matrices were homogenized by a knife mill Grindomix GM-200 (Retsch, Dusseldorf, Germany). In total, 0.5 g of homogenized sample was weighed and extracted with a mixture of water (5 mL), acetone (10 mL), hexane (7.5 mL), and 1.5 g of sodium chloride. The sample was shaken using a standardized device, Agytax (Lab Service Analytica srl, Anzola dell’Emilia, Italy), with the following settings: amplitude 180 mm, velocity 2.0 m/s, acceleration 45 m/s², jerk level 7, delay 0.05 s, and duration 900 s. Subsequently, the sample was shaken for 30 min and centrifuged at 3550× *g* for 5 min. The supernatant (5 mL) was concentrated to 1 mL under nitrogen using a Reacti-Vap evaporation unit and injected into the GC-MS for analysis.

For chromatographic analysis, a Trace GC 2000 gas chromatograph coupled to a DSQ single-quadrupole mass spectrometer (Thermo Finnigan, Bremen, Germany) was used. The extract (1 µL) was injected in splitless mode, setting the injector port temperature at 250 °C. Chromatographic separation was performed using a DB-5 MS 30 m × 0.25 mm × 0.25 mm capillary column (J&W, Agilent, Santa Clara, CA, USA). The column oven was set at 98 °C; after the temperature was increased to 160 °C at a heating rate of 25 °C min^−1^, it was raised to 210 °C using a heating ramp of 4 °C min^−1^. Finally, the temperature was increased to 280 °C at 10 °C/min^−1^ and maintained for 15 min. The helium working flow was set at 1 mL min^−1^. The mass spectrometer transfer line and the ionization source temperature were set at 250 °C and 260 °C, respectively. The electron ionization was set at 70 eV, and the scan time was 100 scan/s. The analyses were conducted in full-scan and selected ion monitoring (SIM) mode.

The acquisition in full-scan mode was carried out in the range of 50–650 *m*/*z,* while the selected ions for the SIM acquisition were 198, 121, and 105 *m*/*z*. A screening analysis was performed in full-scan mode, and the DNOC identification was performed by the NIST Library. The samples that tested positive were then subjected to a confirmatory analysis in SIM mode. A DNOC standard solution at a concentration of 50 mg/kg was used for the quantification. The DNOC reference standard was obtained from HPC Standards GmbH (Borsdorf, Germany). The limit of detection (LOD) was 0.25 mg/kg.

## 3. Results

In total, 58 samples (8.75%) were confirmed to be positive for DNOC out of the 663 analyzed (Table 1).

The determination of DNOC was obtained using gas chromatography coupled to mass spectrometry (GC-MS) with both full-scan and single ion monitoring (SIM) modes (ion values *m*/*z* 198, 121, and 105) (Figure S1).

**Figure 1:**
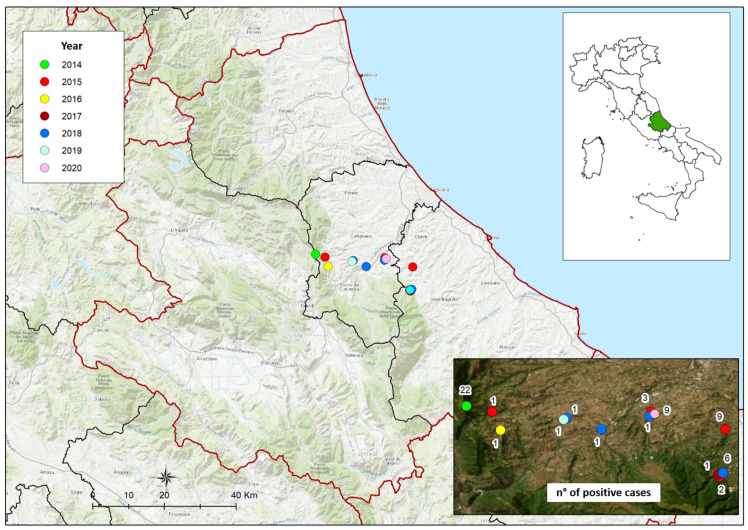
Distribution of DNOC positive cases in the Abruzzo region, Italy.

The positive cases were represented by 49 baits and nine gastric content samples obtained from two red foxes (*Vulpes vulpes*) and seven domestic dogs. All positive samples came from a limited area of the Abruzzo region that is referred to as an area with a particular abundance of truffle grounds (Figure 1).

## 4. Discussion

Intentional and illegal animal poisoning using bait is a global problem, affecting several European countries. In Extremadura (Western Spain), between 2002 and 2018, 246 baits were analyzed, including 32 commercial chemical products used in their preparation. Anticholinesterase compounds, specifically organophosphates and carbamates, were the most frequently detected substances, found in 85.3% of the positive samples. Additionally, 8% of these positive baits contained more than one toxic substance [13].

In Southern Italy, a retrospective study on baits submitted for toxicological analysis at the Istituto Zooprofilattico Sperimentale del Mezzogiorno (IZSM) from 2013 to 2017 revealed that the molluscicide metaldehyde was the most commonly detected substance (63.9%), followed by organochlorine insecticides (29.2%), organophosphine insecticides (11.1%), and anticoagulant rodenticides (9.7%) [14].

In Greece, data collected from 2000 to 2016 documented 1015 poisoning incidents in rural areas, resulting in the deaths of 3248 animals. In 58.7% of the cases, the motive was unknown; however, in the remaining cases, conflicts between humans and wildlife, as well as retaliatory actions among different stakeholders (e.g., hunters versus livestock breeders), were identified as the primary reasons for using poison baits [15].

In Portugal, between January 2014 and October 2020, pesticide residues were found in 239 out of 503 analyzed samples. The most frequently detected pesticide categories were molluscicides (47%) and carbamates (24%), followed by rodenticides (13%) and strychnine (11%), despite its ban in Portugal since 1988. Organophosphates accounted for only 5% of positive cases. The study highlighted that many positive samples involved banned pesticides (e.g., aldicarb and strychnine), while others were linked to commercially available products (e.g., methiocarb and anticoagulant rodenticides) [16].

Comprehensive information on animal poisoning in Central Italy remains scarce and fragmented, with most recent toxicological data coming from the northern regions of the country. A study investigating factors associated with the risk of canine poisoning in Central Italy identified anticoagulant rodenticides, organophosphate pesticides, metaldehyde, and strychnine as the most frequent causes of intoxication. Additionally, territorial characteristics significantly influenced both the frequency and nature of the substances involved. Poisonings by rodenticides and metaldehyde were more common in seashore areas, while organophosphate pesticide, metaldehyde, and strychnine poisonings were more prevalent in hill country areas. Mountain areas were primarily associated with strychnine poisonings [17].

In the northeastern regions of Italy, 642 out of 1831 (35.1%) reported animal poisoning cases were confirmed, and toxic agents were detected in 292 out of 698 (41.8%) analyzed baits. Carbamate insecticides were identified as the leading cause of animal poisonings, while anticoagulant rodenticides were the most frequently found toxicants in poisoned baits [18].

In a separate study of acute poisonings in animals in Northwest Italy, poisoning was identified as the cause of death in about 4% of necropsied animals (356 out of 9512). Domestic pets (9.5%) and synanthropic animals (12.2%) were the most commonly affected. Furthermore, 294 out of 728 baits (40.4%) tested positive for toxic substances and/or inert hazardous material. This study also confirmed that pesticide poisonings remain the most commonly reported in Italy, with rodenticides detected about four times more often in animals than in baits. Interestingly, more than half of the detected insecticides were restricted substances [10].

Another study investigating the types of pesticides involved in domestic and wild animal poisoning in Northern Italy found that 2006 out of 4606 (43.55%) samples tested positive for pesticides. Insecticides, primarily acetylcholinesterase inhibitors (carbamates 17.55%, n = 352; organophosphates 15.15%, n = 304) and organochlorines (29.21%, n = 586), were the most common pesticides involved in intoxications in both domestic and wild animals. This was followed by rodenticides (anticoagulant rodenticides 21.09%, n = 423; zinc phosphide 2.59%, n = 52; chloralose 0.95%, n = 19; and thallium 0.15%, n = 3) and molluscicides (metaldehyde 6.63%, n = 133) [1].

A study by Avolio et al. (2021) [19] on animal poisoning in the Liguria region (Northwest Italy) found that 43.2% of animal poisoning cases were confirmed by toxicological analysis, while toxic agents were detected in 31.1% of baits. Dogs and cats were the most affected species, followed by synanthropic birds. Only 4% of the total poisoning events analyzed involved wild animals, and cases of livestock poisoning were minimal. The primary cause of poisoning and the most commonly detected substances in baits were anticoagulants, while cholinesterase inhibitors, organochlorine pesticides, and carbamates were detected in fewer cases.

In Southeastern Italy, Chirizzi et al. (2020) [9] found that 23.7% of animals (408 out of 1719) and 47.3% of baits (224 out of 474) tested positive for toxic substances. Small animals were positive in 26.0% of cases (341 out of 1310), with metaldehyde, a molluscicide, being the most commonly involved substance. Only 11.4% (16 out of 123) of farm animals tested positive, primarily for rodenticides and pesticides. Among wild animals, 18.5% (53 out of 286) were positive, mainly for pesticides. Metaldehyde was the leading cause of poisoning (42.4%), followed by pesticides (29.7%), rodenticides (26.5%), and other substances (ethylene glycol and metals, 1.2%). Suspected poisoning baits tested positive in 47.3% of cases, with metaldehyde again being the primary substance involved (49.3%), followed by pesticides (26.9%) and rodenticides (23.3%). No cases of herbicide poisoning were found in that study, confirming that herbicides are less commonly involved in animal poisonings [9].

In terms of herbicides, paraquat and fungicides were responsible for 4.4% of all positive cases in Northern Greece between 1994 and 1997 [20]. In Italy, paraquat has been commonly found in baits mixed with strychnine, while in Spain, occasional cases of paraquat toxicity have been reported in dogs in the Murcia region [21]. Herbicides are frequently suspected but rarely confirmed as causes of pet poisonings in France. One of the most widely used herbicides, glyphosate, has an only minimal potential to poison animals, although surfactants used in its liquid formulation, such as polyoxyethylene amine, may be toxic [22].

DNOC is a cresol derivative applied mainly for the preharvest desiccation of potatoes and leguminous seed crops and for the control of broad-leaf weeds in cereals. DNOC has also been utilized as a dormant spray insecticide, particularly for thinning fruit-tree blossoms or killing locusts [23]. DNOC as a chemical occurs as a yellow prismatic solid that is sparingly soluble in water. It was classified as class Ib, “highly hazardous”, in the WHO Recommended Classification of Pesticides by Hazards [24].

The Lethal Dose 50 (LD50) value of DNOC in rats ranges from 20 to 85 mg/kg body weight (b.w.), 50 to 100 mg/kg b.w. in pigs, and 50 mg/kg in domestic cats [25,26].

Although the use of DNOC as a pesticide has been prohibited in Europe since 1991, significant amounts of unused pesticides are still available [24]. So, the poisoning of animals by this banned compound could still happen, as demonstrated in the present study. Moreover, the incidence of clinical poisoning by DNOC could be also underestimated, since this chemical is not often included in the standard analytical procedures applied in cases of the suspected poisoning of animals.

**Figure 2:**
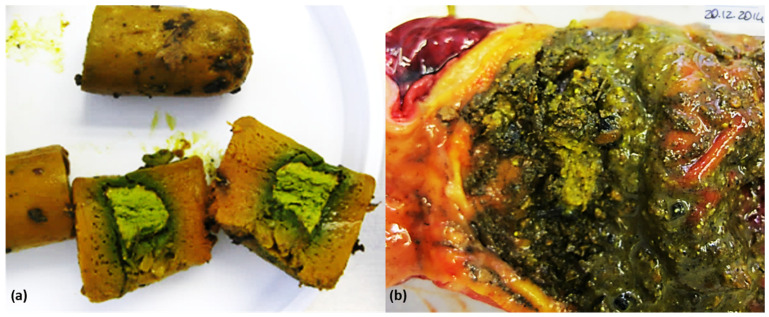
Poison bait composed of meat products with the chemical substance placed in the center (**a**) and gastric content of a domestic dog (**b**) with DNOC.

According to our knowledge, this is the first report of detection of DNOC in poisoned animals in Italy. The anatomopathological alterations of animals poisoned with DNOC were greatly unspecific, and in most cases, the only observed finding was the fur and the stomach content of the animal being yellow (Figure 2).

This evidence was verified by other authors [27] in a dog that had been poisoned. In addition, the yellow color of the hands, nails, and hair, as well as the greenish-yellow color of the conjunctiva, has been described in cases of human intoxication [28]. In our case, though, there was no record that poisoned animals had developed any signs of disease. However, DNOC is an uncoupler of oxidative phosphorylation; as a consequence, clinical signs of acute toxicity in humans and experimental animals are related to increased basal metabolic rates, such as profuse sweating, increased pulse and respiratory rates, lethargy, headache, nausea, collapse, and coma [24]. Mean concentrations detected were high and of potential toxicological clinical concern. The availability of toxicological data for DNOC in domestic animals is limited. Experimental studies in cats showed death following inhalation exposure to DNOC liquid or solid aerosols for 4 h at 40 and/or 100 mg/m^3^, and in other cats exposed to DNOC liquid aerosol 4 h/day for 1 month at 2 mg/m^3^ [28].

All positive cases came from a limited area of the Abruzzo region that is referred to as an area with a particular abundance of truffle grounds. Such evidence indicates the intentional nature of poisoning cases. However, if animals are accidentally sprayed or have immediate access to forage which has been sprayed, they may also be poisoned [23]. It is interesting to note that positive cases between 2014 and 2020 have been identified, with no positive case in 2021 or 2022, indicating the effectiveness of the applied strategies for fighting illegal poisonings and minimizing their impact on animal health.

## 5. Conclusions

In 2019, the Italian Ministry of Health instituted the National Portal of Intentional Animal Poisoning. This portal aims to provide complete digital management of reported suspected cases of animal poisoning in accordance with current regulations and to enable constant monitoring of the phenomenon, including its temporal and spatial characteristics. The goal is to provide useful information to both citizens and law enforcement authorities for the prevention and suppression of this issue. Constant updates to this system, as well as the integration of new chemical substances into the analytical procedures used in diagnosing animal poisoning, are essential to control and counteract this phenomenon.

In this study, we demonstrated for the first time that the poisoning of domestic and wild animals by the banned pesticide DNOC still occurs in Italy. Despite the prohibitions on the use of several pesticides in the European Union and Italy, acute poisoning continues to affect domestic animals and wildlife. To gain a better understanding of the incidence of DNOC poisoning in domestic and wild animals in Italy, it is necessary to conduct more detailed studies across the country, as this study focuses on a specific geographical area.

## Figures and Tables

**Table 1:** Number of analyzed samples between 2014 and 2022, number of positive cases for each year, and quantified mean DNOC concentrations.

Year	N° of Positive Cases/ Total Analyzed Samples (% of Positive)	Type of Positive Samples	Mean Concentration (min-max) (g/kg)
2014	22/26 (85%)	5 gastric content samples, 17 baits	-
2015	15/30 (50%)	2 gastric content samples, 13 baits	6.2 (0.88–10)
2016	1/7 (14%)	1 bait	15.36
2017	1/2 (50%)	1 bait	12.69
2018	9/16 (56%)	1 gastric content sample, 8 baits	6.64 (0.73–13.23)
2019	1/2 (50%)	1 gastric content sample	5.1
2020	9/9 (100%)	9 baits	11.1 (1.78–22.99)
2021	0/329 (0%)	-	-
2022	0/242 (0%)	-	-
Total	58/663		

## Data Availability

The original contributions presented in this study are included in the article and in the Supplementary Material; further inquiries can be directed to the corresponding author.

## Supplementary Materials

The following supporting information can be downloaded at: https://www.mdpi.com/article/10.3390/ani14172483/s1, Figure S1: GC-MS chromatogram showing the chemical analysis.

## References

[1] Bertero A., Chiari M., Vitale N., Zanoni M., Faggionato E., Biancardi A., Caloni F. (2020). Types of pesticides involved in domestic and wild animal poisoning in Italy. STOTEN.

[2] Boye K., Lindström B., Boström G., Kreuger J. (2019). Long-term data from the Swedish national environmental monitoring program of pesticides in surface waters. J. Environ. Qual..

[3] Kazimierczak R., Średnicka-Tober D., Golba J., Nowacka A., Hołodyńska-Kulas A., Kopczyńska K., Gnusowski B. (2022). Evaluation of pesticide residues occurrence in random samples of organic fruits and vegetables marketed in Poland. Foods.

[4] Merola C., Fabrello J., Matozzo V., Faggio C., Iannetta A., Tinelli A., Crescenzo G., Amorena M., Perugini M. (2022). Dinitroaniline herbicide pendimethalin affects development and induces biochemical and histological alterations in zebrafish early-life stages. STOTEN.

[5] Chiari M., Cortinovis C., Vitale N., Zanoni M., Faggionato E., Biancardi A., Caloni F. (2017). Pesticide incidence in poisoned baits: A 10-year report. STOTEN.

[6] Wang Y., Kruzik P., Helsberg A., Helsberg I., Rausch W.D. (2007). Pesticide poisoning in domestic animals and livestock in Austria: A 6 years retrospective study. For. Sci. Int..

[7] Caloni F., Cortinovis C., Rivolta M., Davanzo F. (2012). Animal poisoning in Italy: 10 years of epidemiological data from the Poison Control Centre of Milan. Vet. Rec..

[8] Caloni F., Cortinovis C., Rivolta M., Davanzo F. (2016). Suspected poisoning of domestic animals by pesticides. STOTEN.

[9] Chirizzi D., Manca R., Summa S., Pacioll I., Toce M., Romano A., Muscarella M. (2020). Suspected veterinary poisoning cases: A retrospective toxico-epidemiology study (2009–2019) in south-eastern Italy. TiM.

[10] Di Blasio A., Bertolini S., Gili M., Avolio R., Leogrande M., Ostorero F., Zoppi S. (2020). Local context and environment as risk factors for acute poisoning in animals in northwest Italy. STOTEN.

[11] Ruiz-Suárez N., Boada L.D., Henríquez-Hernández L.A., González-Moreo F., Suárez-Pérez A., Camacho M., Luzardo O.P. (2015). Continued implication of the banned pesticides carbofuran and aldicarb in the poisoning of domestic and wild animals of the Canary Islands (Spain). STOTEN.

[12] Italian Ministerial Decree of 18 December 2008—Italian Official Journal no. 13, 17 January 2009. https://www.izsvenezie.it/documenti/temi/avvelenamenti/provvedimenti/2008-12-18-ordinanza.pdf.

[13] Ibáñez-Pernía Y., Hernández-Moreno D., Pérez-López M., Soler-Rodríguez F. (2022). Use of poisoned baits against wildlife. A retrospective 17-year study in the natural environment of Extremadura (Spain). Environ. Pollut..

[14] De Roma A., Miletti G., D’Alessio N., Marigliano L., Bruno T., Gallo P., Esposito M. (2018). Inspective and toxicological survey of the poisoned baits and bites. Forensic Sci. Int..

[15] Ntemiri K., Saravia V., Angelidis C., Baxevani K., Probonas M., Kret E., Xirouchakis S.M. (2018). Animal mortality and illegal poison bait use in Greece. Environ. Monit. Assess..

[16] Grilo A., Moreira A., Carrapiço B., Belas A., São Braz B. (2021). Epidemiological study of pesticide poisoning in domestic animals and wildlife in Portugal: 2014–2020. Front. Vet. Sci..

[17] Calzetta L., Roncada P., Piras C., Soggiu A., Liccardi G., Mattei M., Pistocchini E. (2018). Geographical characteristics influencing the risk of poisoning in pet dogs: Results of a large population-based epidemiological study in Italy. TVJ.

[18] Bille L., Toson M., Mulatti P., Dalla Pozza M., Capolongo F., Casarotto C., Binato G. (2016). Epidemiology of animal poisoning: An overview on the features and spatio-temporal distribution of the phenomenon in the north-eastern Italian regions. Forensic Sci Int.

[19] Avolio R., Andreoli T., Ercolini C., Mignone W., Beltrame R., Razzuoli E., Dellepiane M. (2021). Retrospective data analysis of animal poisoning events in Liguria. Vet Anim Sci.

[20] Antoniou V., Zantopoulos N., Tsitsamis S. (1998). Poisoning with substances in dogs and cats: A report of 129 cases. Anima.

[21] Motas-Guzmán M., Marla-Mojica P., Romero D., Martinez-Lopez E., García-Fernández A.J. (2003). Intentional poisoning of animals in southeastern Spain: A review of the veterinary toxicology service from Murcia, Spain. Vet Hum Toxicol.

[22] Berny P., Caloni F., Croubels S., Sachana M., Vandenbroucke V., Davanzo F., Guitart R. (2010). Animal poisoning in Europe. Part 2: Companion animals. TVJ.

[23] (2000). Environmental Protection Agency (EPA). https://www.epa.gov/sites/default/files/2016-09/documents/4-6-dinitro-o-cresol.pdf.

[24] (2000). World Health Organization (WHO). https://iris.who.int/bitstream/handle/10665/42283/WHO_EHC_220.pdf?sequence=1&isAllowed=y.

[25] (2018). Agency for Toxic Substances and Disease Registry (ATSDR). https://www.atsdr.cdc.gov/ToxProfiles/tp63.pdf.

[26] Gig. Tr. Prof. Zabol. (V/O Mezhdunarodnaya Kniga, 113095 Moscow, USSR) 1965, V.1-36, 1957–1992. https://www.umweltbundesamt.de/sites/default/files/medien/publikation/long/3414.pdf.

[27] Đurđević B., Samojlović M., Kartalović B., Ratajac R., Pelić R., Pajić M., Polaček V. (2018). Poisoning of domestic carnivores by banned pesticides in South Bačka district. AVM.

[28] Burkatskaya E.N. (1965). The toxicity of dinirro-o-cresol for warm-blooded animals and problems of its use for industrial hygiene. Gig. Sanit..

