# Supplementary figures and images for: The Determination of the Prohibited Herbicide 4,6-Dinitro-Ortho-Cresol (DNOC) in Poisoned Domestic and Wild Animals in Italy
Source: Animals (Basel). 2024 Aug 26;14(17):2483. doi: 10.3390/ani14172483 (PMC11394050; doi:10.3390/ani14172483)

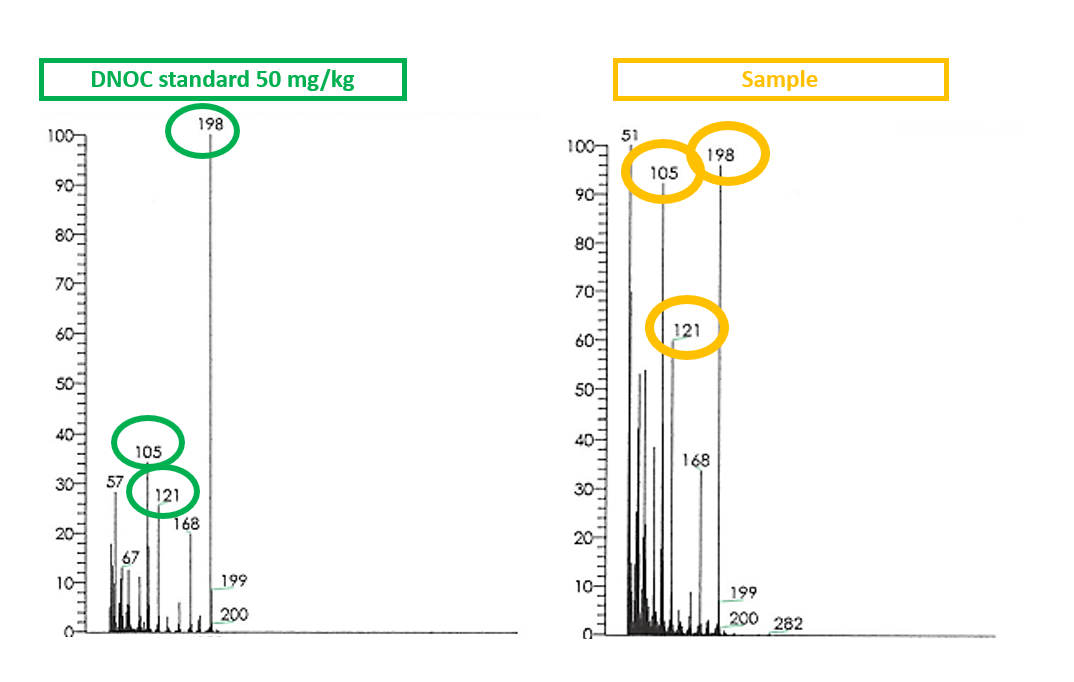

Supplement: Supplementary file 1 [file animals-14-02483-s001.zip › animals-3179532-supplementary.tif]
